# Comparative overall survival of CDK4/6 inhibitors in combination with endocrine therapy in advanced breast cancer

**DOI:** 10.1038/s41598-024-53151-8

**Published:** 2024-02-07

**Authors:** Coralea Kappel, Mitchell J. Elliott, Vikaash Kumar, Michelle B. Nadler, Alexandra Desnoyers, Eitan Amir

**Affiliations:** 1 Princess Margaret Cancer Centre, University of Toronto, Toronto, ON Canada; 2 Hôpital Charles-Lemoyne, Greenfield Park, QC Canada; 3 Princess Margaret Cancer Centre, 610 University Ave, 700U, 7-721, Toronto, ON M5G 2M9 Canada

**Keywords:** Breast cancer, Targeted therapies

## Abstract

Individual trials of abemaciclib, palbociclib, and ribociclib show a similar impact on progression-free survival yet differing statistical significance for overall survival (OS). A robust comparative evaluation of OS, safety, and tolerability of the three drugs is warranted. A systematic literature search identified phase 3 randomized clinical trials reporting OS of CDK4/6 inhibitors (CDK4/6i) in combination with endocrine therapy in ER-positive/HER2-negative advanced breast cancer. Trial-level data on OS and common and serious adverse events (AE) were extracted for each drug. In the absence of direct comparisons, a network meta-analysis was performed to evaluate pairwise comparative efficacy, safety, and tolerability of each of the CDK4/6i. Seven studies comprising of 4415 patients met the inclusion criteria. Median follow-up was 73.3 months (range: 48.7–97.2 months). There were no statistically significant differences in OS between any of the CDK4/6i. Compared to palbociclib, ribociclib and abemaciclib both showed significantly higher GI toxicity (grade 1–2 vomiting OR 1.87 [95% CI 1.37–2.56] and OR 2.27 [95% CI 1.59–3.23] respectively). Compared to palbociclib, abemaciclib was associated with more grade 3–4 diarrhea OR 118.06 [95% CI 7.28–1915.32]. In contrast, palbociclib was associated with significantly more neutropenia than ribociclib and abemaciclib but significantly lower risk of grade 3–4 infections. Abemaciclib had significantly less grade 3–4 transaminitis and grade 3–4 neutropenia than ribociclib. Treatment discontinuation and death due to AE were significantly higher with abemaciclib than palbociclib and ribociclib. There is no statistically significant difference in OS between CDK4/6i despite differing statistical significance levels of individual trials. Real-world data analyses may help to identify if there is a meaningful inter-drug difference in efficacy. Significant differences between CDK4/6i are observed for safety and tolerability outcomes.

## Introduction

Inhibition of cyclin dependent kinase 4 (CDK4) and cyclin dependant kinase 6 (CDK6) in combination with endocrine therapy is the first-line standard of care for hormone receptor positive and erb-B2-negative (HR+/HER2−) locally advanced or metastatic breast cancer (MBC)^1–3^. In phase III trials, CDK4/6 inhibitors trials, palbociclib, ribociclib, and abemaciclib have shown a consistent improvement in progression-free survival (PFS) when combined with an aromatase inhibitor (AI), fulvestrant, or tamoxifen^4,5^ with the hazard ratios for PFS ranging between 0.50 and 0.59. In contrast, while individual trials for ribociclib with endocrine therapy have reported statistically significant improvement in overall survival (OS), such improvements have not been reported for palbociclib or abemaciclib^6–11^. This is reflected in the National Comprehensive Cancer Network guidelines where ribociclib is the only category 1 preferred first-line treatment option for HR+/HER2− MBC in combination with an AI; whereas both abemaciclib and ribociclib are category 1 preferred first-line in combination with fulvestrant^3^.

CDK4/6 inhibitors can be associated with significant symptom burden that may limit tolerability and impact patients’ health-related quality of life^12^. In a pooled analysis of clinical trials, more than 70% of older patients had their treatment dose reduced and more than 15% discontinued treatment^13^. Tolerability is a key metric for CDK4/6 inhibitors given the duration of treatment can extend over 2 years, especially when used in the first-line setting.

A robust analysis of both relative efficacy and relative tolerability is therefore of interest to help clinicians and patients make informed decisions about the optimal agent to be used.

## Methods

### Search strategy and study selection

A network meta-analysis, registered in PROSPERO (registration number CRD42023392416) was performed in accordance with the Preferred Reporting Items for Systematic Reviews and Meta-analyses guidelines (PRISMA)^14^. Inclusion criteria comprised phase 3 randomized controlled trials (RCTs) in which patients with HR+/HER2− metastatic breast cancer were treated with a CDK4/6 inhibitor in combination with endocrine therapy (AI, fulvestrant or tamoxifen) compared to endocrine therapy alone in the first or second-line setting. There was no limitation on year or language of publication. Meta-analyses, single-arm trials, and observational studies were excluded. Only studies of human subjects were included. When more than one publication was identified for the same clinical trial, data from the most recent or complete report were included.

A search strategy was constructed using ClinicalTrials.gov. Titles and abstracts identified by these strategies were screened independently by two reviewers (C.K. and E.A.) for inclusion; disagreements were resolved by consensus. The following variables from all eligible manuscripts were extracted: year of publication, median duration of follow-up, study sample size and the treatment in the experimental and control groups. For each approved CDK4/6 inhibitor, data was extracted on efficacy and on pre-specified common and serious treatment related adverse events. For efficacy outcomes, the study-reported hazard ratios (HR) and respective 95% confidence intervals (CI) for overall survival (OS) were extracted. For safety and tolerability, the data extracted included treatment-related death, treatment discontinuation due to adverse event and selected adverse events (AEs). For hematological toxicities, data were extracted on grade 3–4 neutropenia, anemia, and thrombocytopenia. For GI toxicities, data were extracted on both grade 1–2 and grade 3–4 diarrhea, nausea, and vomiting. Additional data was extracted on grade 1–2 stomatitis, grade 1–2 fatigue and/or asthenia, grade 3–4 venous thromboembolism (VTE), grade 3–4 transaminitis, grade 3–4 dyspnea and/or cough, grade 3–4 infection, grade 3–4 prolonged QT and grade 1–2 alopecia. The number of events and the number of patients at risk were extracted individually for both the CDK4/6 inhibitor and control groups in each trial. Outcome measures were obtained from the most recently published manuscripts and cross-referenced with data in the clinicaltrials.gov registry to ensure consistency.

### Data synthesis and statistical analysis

When more than one study reported data for either efficacy or safety and tolerability outcomes, these were pooled in a meta-analysis using RevMan 5.4 (Cochrane Collaboration, Copenhagen, Denmark). For efficacy, HR for OS and associated 95% CI were pooled using generic inverse variance. For toxicity profile, the odds ratio (OR) and associated standard error (SE) for each adverse event were calculated relative to endocrine therapy alone using the Mantel–Haenszel method. Pooling was performed using fixed effects modeling irrespective of statistical heterogeneity. Due to the expected differences in endocrine therapy and patient characteristics between studies, analyses were performed separately for each endocrine therapy backbone (AI/tamoxifen or fulvestrant) to compare ribociclib and abemaciclib to palbociclib. Then a network meta-analysis was performed using WINBUGS within Microsoft Excel (Microsoft Corp, Redmond WA). A post-hoc sensitivity meta-analysis was also performed repeating the analysis utilizing post-hoc data from one trial in which there were substantial missing data. Statistical tests were two-sided, and statistical significance was defined as p < 0.05. No correction was made for multiple statistical testing.

### Ethics approval

This study was exempt from ethics board approval since it used publicly available data exclusively.

## Results

**Figure 1:**
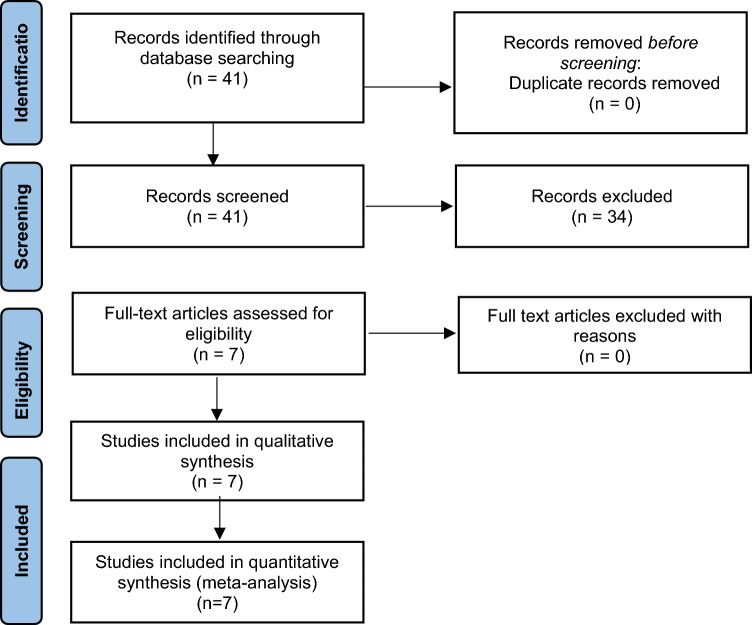
PRISMA flow diagram.

The study selection schema is shown in Fig. 1. Seven phase III RCTs were included in the analysis including PALOMA-2, PALOMA-3, MONALEESA-2, MONALEESA-3, MONALEESA-7, MONARCH-2, and MONARCH-3^6–11,15,16^. In total, the analysis comprised of 4415 patients, of which 2718 patients received a CDK4/6 inhibitor (1153 ribociclib, 791 palbociclib,774 abemaciclib). In 4 RCTs (1441 patients), the endocrine therapy backbone was an AI or tamoxifen and in 3 RCTs (1277 patients) it was fulvestrant. The median follow-up was 70.2 months (range: 48.7–97.2 months). Characteristics of the studies are outlined in Table 1.

**Table 1:** Characteristics of included studies.

Study characteristics	CDK4/6i with AI or tamoxifen			CDK4/6i with fulvestrant			
	PAL-2	MONALEESA-2	MONALEESA-7	MONARCH-3	PALOMA-3	MOLANEESA-3	MONARCH 2
Year of initial publication	2015	2016	2018	2017	2016	2018	2017
Year of updated data	2022	2022	2022	2023	2021	2021	2020
Total number of patients	666	668	672	493	521	726	669
Line	1st	1st	1st and 2nd line (after chemotherapy)	1st	Progression after ET (adjuvant or 1st line)	1st and 2nd line	Progression after ET (neo/adjuvant or 1st line)
Menopausal status	Post	Post	Pre	Post	Pre/post	Post	Pre/post
Median follow-up (months)	90	80	53.5	97.2	73.3	56.3	48.7
CDK4/6i	Palbociclib	Ribociclib	Ribociclib	Abemaciclib	Palbociclib	Ribociclib	Abemaciclib
Median OS in placebo + endocrine arm (months	51.2	51.4	48.0	54.5	28	41.5	37.3
Median OS in CDK4/6I + endocrine arm (months	53.9	63.9	58.7	66.8	34.8	53.7	46.7
Reported HR for OS	0.956	0.76	0.76	0.804	0.814	0.73	0.757
Reported 95% CI for HR of OS	0.777–1.177	0.63–0.93	0.61–0.96	0.637–1.015	0.644–1.029	0.59–0.90	0.606–0.945

### Efficacy

In the meta-analysis of the CDK4/6 inhibitors with an AI backbone, palbociclib had a non-significantly worse OS compared to ribociclib and abemaciclib (HR 1.26 [95% CI 0.88–1.80, p = 0.21] and 1.19 [95% CI 0.80–1.76, p = 0.39]) respectively. There were no differences in OS with ribociclib compared to abemaciclib (HR 1.06 [95% CI 0.80–1.41, p = 0.70]). For the fulvestrant backbone, palbociclib had similar OS compared to both ribociclib and abemaciclib; HR 1.12 (95% CI 0.75–1.66, p = 0.59) and 1.08 (95% CI 0.72–1.61, p = 0.73) respectively. Similarly, there were no differences in OS between ribociclib and abemaciclib (HR 0.96 [95% CI 0.66–1.42, p = 0.85]). Table 2 summarizes all indirect comparisons between the 3 different CDK4/6 inhibitors (forest plots for these analyses are shown in the Supplementary File.

**Table 2:** Differences in OS between the CDK4/6i with any ET or AI backbone with the PALOMA-2 sensitivity analysis.

AI backbone			
Control	Palbociclib	Ribociclib	Abemaciclib
AI backbone			
Palbociclib	–	0.79 (0.56, 1.14), p = 0.21	0.84 (0.57, 1.24), p = 0.39
Ribociclib	1.26 (0.88, 1.80), p = 0.21	–	1.06 (0.80, 1.41), p = 0.70
Abemaciclib	1.19 (0.80, 1.76), p = 0.39	0.95 (0.71, 1.26), p = 0.70	–
Fulvestrant backbone			
Palbociclib	–	0.90 (0.60, 1.33), p = 0.59	0.93 (0.62, 1.40), p = 0.73
Ribociclib	1.12 (0.75, 1.66), p = 0.59	–	1.04 (0.71, 1.52), p = 0.85
Abemaciclib	1.08 (0.72, 1.61), p = 0.73	0.96 (0.66, 1.42), p = 0.85	–

PALOMA-2 sensitivity analysis			
Control	Palbociclib	Ribociclib	Abemaciclib
Palbociclib	–	0.87 (0.61, 1.25), p = 0.46	0.93 (0.63, 1.37), p = 0.70
Ribociclib	1.14 (0.80, 1.63), p = 0.46	–	1.06 (0.80, 1.41), p = 0.70
Abemaciclib	1.08 (0.73, 1.60), p = 0.70	0.95 (0.71, 1.26), p = 0.70	–

HR (95% CI), p value.

In the PALOMA-2 trial, OS data was missing in 13% of the participants in the experimental arm and 21% in the control arm. In the post-hoc analysis utilizing data from the PALOMA-2 trial which excluded missing data, there was a smaller magnitude association with worse OS with palbociclib compared to ribociclib and abemaciclib (HR 1.14 [95% CI 0.80–1.63, p = 0.46] and 1.08 [95% CI 0.73, 1.60 p = 0.70] respectively). This lower magnitude effect remained statistically non-significant.

### Safety and tolerability

Differences in safety and tolerability were observed between the 3 different CDK4/6 inhibitors (see Table 3). When assessing the AI/tamoxifen backbone, compared to palbociclib, abemaciclib had significantly more GI toxicity including more grade 1–2 vomiting and grade 1–2 diarrhea. Grade 3–4 neutropenia was significantly lower with abemaciclib however grade 3–4 infections were significantly higher. Grade 3-transaminitis was also higher with abemaciclib. Compared to palbociclib, ribociclib had significantly more GI toxicity with more grade 1–2 nausea, more grade 1–2 vomiting, grade 3–4 vomiting and grade 3–4 transaminitis. In comparison to ribociclib, abemaciclib had significantly more diarrhea of any grade and more grade-3–4 anemia. When assessing the fulvestrant backbone, compared to palbociclib, abemaciclib had significantly more GI toxicity including all grade nausea, grade 1–2 vomiting, grade 1–2 vomiting, grade 3–4 diarrhea. Abemaciclib had less grade 3–4 neutropenia than palbociclib but more grade 3–4 infections. Furthermore grade 3–4 dyspnea/pneumonitis was higher with abemaciclib. Compared to palbociclib, ribociclib had significantly more grade 3–4 QT prolongation and grade 3–4 transaminitis. Furthermore, ribociclib had more GI toxicity than palbociclib including more grade 1–2 nausea, grade 1–2 vomiting, and grade 1–2 diarrhea. Ribociclib had less grade 1–2 fatigue/asthenia than palbociclib, less grade 3–4 neutropenia, but more grade 3–4 infections.

**Table 3:** Adverse events between the CDK4/6i with any ET or AI backbone.

	With AI			With fulvestrant		
	OR	95% CI	P value	OR	95% CI	P value
Abemaciclib vs palbociclib (control)						
Anemia grade 3–4	1.32	0.73–2.39	0.352	**3.34**	1.65–6.78	0.001
Neutropenia grade 3–4	**0.17**	0.13–0.24	< 0.001	**0.23**	0.17–0.31	< 0.001
Neuropathy grade 3–4	0.45	0.02–11.12	0.627	3.93	0.19–82.14	0.376
Prolonged QT grade 3–4	0.45	0.02–11.12	0.627	0.78	0.05–12.54	0.859
Transaminitis grade 3–4	**7.55**	2.57–22.21	< 0.001	2.54	1.0–6.44	0.050
Nausea grade 1–2	1.25	0.93–1.67	0.139	**1.81**	1.35–2.42	< 0.001
Nausea grade 3–4	5.49	0.61–49.32	0.129	**20.11**	1.19–340.89	0.038
Vomiting grade 1–2	**2.27**	1.59–3.23	< 0.001	**1.95**	1.37–2.78	0.000
Vomiting grade 3–4	3.43	0.66–17.8	0.143	3.15	0.35–28.3	0.306
Diarrhea grade 1–2	**7.56**	5.48–10.44	< 0.001	**9.69**	6.95–13.49	< 0.001
Diarrhea grade 3–4	**7.65**	3.15–18.55	< 0.001	**118.06**	7.28–1915.32	0.001
Stomatitis grade 1–2	0.81	0.53–1.23	0.330	**1.52**	1.01–2.29	0.045
Alopecia grade 1–2	0.78	0.57–1.07	0.121	1.03	0.71–1.5	0.876
Fatigue/asthenia grade 1–2	1.18	0.88–1.58	0.269	1.09	0.81–1.45	0.569
Dyspnea/pneumonitis grade 3–4	1.92	0.6–6.11	0.272	**11.28**	1.48–86.2	0.019
Infection grade 3–4	**8.54**	3.27–22.32	< 0.001	**4.61**	1.76–12.07	0.002
VTE grade 3–4	3.11	0.95–10.2	0.061	1.78	0.54–5.82	0.343
Discontinuation due to AE	**1.84**	1.2–2.83	0.005	**2.49**	1.34–4.64	0.004
Treatment-related death	1.52	0.63–3.6	0.352	15.18	0.88–261.71	0.061
Ribociclib vs palbociclib (control)						
Anemia grade 3–4	0.68	0.38–1.21	0.194	1.37	0.63–2.99	0.427
Neutropenia grade 3–4	**0.4**	0.31–0.51	< 0.001	**0.73**	0.55–0.97	0.039
Neuropathy grade 3–4	0.22	0.01–5.43	0.355	5.03	0.26–97.76	0.286
Prolonged QT grade 3–4	4.01	0.48–33.42	0.199	**11.03**	11.45–83.87	0.020
Transaminitis grade 3–4	**14.73**	5.35–40.52	< 0.001	**8.94**	3.83–20.88	< 0.001
Nausea grade 1–2	**1.34**	1.05–1.72	0.019	**1.63**	1.22–2.17	0.001
Nausea grade 3–4	7.41	0.95–57.57	0.056	10.88	0.62–191.08	0.103
Vomiting grade 1–2	**1.87**	1.37–2.56	< 0.001	**1.71**	1.2–2.42	0.003
Vomiting grade 3–4	**5.42**	1.24–23.67	0.025	5.06	0.62–41.31	0.130
Diarrhea grade 1–2	1.15	0.88–1.51	0.315	**1.45**	1.05–2.01	0.024
Diarrhea grade 3–4	1.11	0.4–3.07	0.841	5.03	0.26–97.76	0.286
Stomatitis grade 1–2	0.83	0.59–1.17	0.288	0.82	0.53–1.27	0.374
Alopecia grade 1–2	**0.76**	0.58–0.98	0.034	1.13	0.79–1.63	0.513
Fatigue/asthenia grade 1–2	1.11	0.86–1.42	0.406	**0.73**	0.54–0.98	0.036
Dyspnea/pneumonitis grade 3–4	1.88	0.67–5.24	0.227	5.79	0.72–46.54	0.099
Infection grade 3–4	**3.84**	1.47–10.01	0.006	**5.64**	2.19–14.51	< 0.001
VTE grade 3–4	1.33	0.4–4.45	0.644	1.99	0.63–6.29	0.241
Discontinuation due to AE	0.8	0.53–1.22	0.300	**2.31**	1.24–4.29	0.008
Treatment-related death	0.59	0.24–1.47	0.257	3.59	0.17–74.97	0.410
Abemaciclib vs ribociclib (control)						
Anemia grade 3–4	**1.95**	1.09–3.49	0.025	**2.44**	1.39–4.27	0.002
Neutropenia grade 3–4	0.44	0.33–0.59	< 0.001	**0.32**	0.24–0.42	< 0.001
Neuropathy grade 3–4	2.04	0.04–130.26	0.737	0.73	0.12–4.38	0.731
Prolonged QT grade 3–4	0.16	0.01–2.77	0.208	**0.07**	0.01–0.54	0.011
Transaminitis grade 3–4	**0.15**	0.31–0.85	0.032	**0.28**	0.17–0.48	< 0.001
Nausea grade 1–2	0.93	0.71–1.22	0.600	1.11	0.86–1.44	0.432
Nausea grade 3–4	0.74	0.23–2.34	0.608	1.9	0.74–4.88	0.182
Vomiting grade 1–2	1.12	0.9–1.63	0.554	1.14	0.85–1.53	0.383
Vomiting grade 3–4	0.63	0.23–1.75	0.375	0.62	0.18–2.14	0.450
Diarrhea grade 1–2	**6.55**	4.87–88	< 0.001	**6.68**	5.01–8.91	< 0.001
Diarrhea grade 3–4	**6.9**	3.34–14.26	< 0.001	**27.16**	8.47–87.14	< 0.001
Stomatitis grade 1–2	0.97	0.65–1.45	0.882	**1.86**	1.26–2.74	0.002
Alopecia grade 1–2	1.02	0.76–1.38	0.898	0.91	0.65–1.27	0.579
Fatigue/asthenia grade 1–2	1.06	0.81–1.4	0.681	**1.49**	1.13–1.96	0.005
Dyspnea/pneumonitis grade 3–4	1.02	0.41–2.56	0.966	1.95	0.81–4.69	0.136
Infection grade 3–4	**2.23**	1.3–3.81	0.003	0.82	0.49–1.36	0.442
VTE grade 3–4	2.34	0.89–6.12	0.083	0.89	0.37–2.8	0.842
Discontinuation due to AE	**2.3**	1.53–3.45	< 0.001	1.08	0.69–1.68	0.733
Treatment-related death	**2.55**	1.05–6.22	0.039	**5.01**	1.08–23.32	0.040

Significant OR bolded.

Compared to ribociclib and palbociclib, abemaciclib had more treatment discontinuation secondary to adverse events. There was no significant difference between ribociclib and palbociclib. Treatment-related death was higher with abemaciclib compared to other CDK4/6 inhibitors (see Table 3). This association was statistically significant for the comparison between abemaciclib and ribociclib and approached but did not meet statistical significance significant for the comparison between abemaciclib and palbociclib.

## Discussion

Three CDK4/6 inhibitors have been approved for use in combination with endocrine therapy for HR+/HER− MBC. While all have shown superiority over endocrine therapy alone, the relative efficacy, safety and tolerability is unknown as no head-to-head trials have been performed. PFS effects have been very consistent for all CDK4/6i trials, with HR ranging between 0.50 and 0.59 and with meta-analyses not suggesting any statistically significant or clinically meaningful differences in PFS between drugs^17^. Therefore, the main markers of differentiation in the efficacy of drugs have been measured by OS. In this study, we performed a network meta-analysis to indirectly evaluate the differences in OS and safety profile of these agents. Our results show that efficacy differences in OS between the three agents are non-significant, and in most cases, effect sizes are not clinically meaningful irrespective of statistical significance. However, as expected, marked differences in safety and tolerability were identified.

While no statistically significant difference in OS was observed between the 3 CDK4/6 inhibitors, there was a non-significant association with shorter OS benefit with palbociclib than the other CDK4/6 inhibitors. The reasons for this are unclear but may reflect trial design rather than inter-drug differences. The OS analysis for PALOMA-2 was limited by a substantial proportion of missing data. OS was missing in 13% of the participants in the experimental arm and 21% in the control arm. In a post-hoc sensitivity analysis of the PALOMA-2 trial excluding participants with missing OS data, larger magnitude relative (HR 0.87 vs 0.96) and absolute effects (difference in median OS 7 vs 2.7 months) were observed. However, as expected, with the loss of power associated with any sensitivity analysis, the effect remained non-significant^15,18^. Using these post-hoc data in our meta-analysis resulted in lower magnitude effects for OS between palbociclib and other CDK4/6 inhibitors. These effects remained non-significant and based on thresholds recommended by the American Society of Clinical Oncology, were of borderline clinical meaningfulness^19^.

Another notable difference between these trials relates to the potential for informative censoring. The difference between study arms in the proportion of patients who were censored for reasons other than end of follow-up (e.g. premature loss to follow up due to AEs or withdrawal of consent) was higher with ribociclib than with palbociclib studies (> 5% in MONALEESA-2 versus < 1% in PALOMA-2). The reasons for unbalanced censoring are unclear, but may impact both the cross-trial comparison of different CDK4/6 inhibitors and meta-analytic comparisons^20,21^.

Consistent with prior reports, substantial differences in safety and tolerability were observed between the different CDK4/6 inhibitors^22^. In general, compared to ribociclib and abemaciclib, palbociclib showed more frequent hematological toxicity, but less frequent gastrointestinal toxicity. Patients with HR+/HER2− MBC report shortness of breath, fatigue, pain and vomiting as the most bothersome symptoms affecting their quality of life^23^. Furthermore, in a meta-analysis of phase 3 breast cancer trials, patients reporting more diarrhea had lower health-related quality of life and worse physical function^24^. Discussing side effect differences between drugs is an important method to increase patients’ satisfaction and increase adherence to treatment^25^. Of note, there were more treatment-related deaths reported with abemaciclib than with other CDK4/6 inhibitors, although this observation was only statistically significant in the comparison of abemaciclib with ribociclib. This finding should be interpreted with caution given that it is possible that these deaths may be related to the breast cancer despite not meeting imaging criteria for progression. It can be difficult to distinguish treatment-related from disease-related causes of death especially among patients with breast cancer who do not have disease which is measurable by Response Evaluation Criteria in Solid Tumors (RECIST) criteria^26^. However, with data suggesting that mechanisms of resistance to CDK4/6 inhibitors seem uniform between the different agents, the higher odds of non-cancer deaths with abemaciclib relative to placebo compared to other CDK4/6 inhibitors is an important observation^27^.

Our study has some limitations. This is a literature-based network meta-analysis rather than using individual patient data. Although the included studies were generally homogenous, there were differences in endocrine therapy backbone, patient populations (e.g. menopausal status) and as detailed above there was concern for post-randomization differences such as missing data and potential for unbalanced informative censoring among some studies. To address inter-study heterogeneity, our analysis compared studies with the same endocrine therapy backbone which would limit heterogeneity, but results in a smaller sample size for comparison and consequently reduced statistical power. There is therefore an incomplete ability to assess the assumptions of transitivity. However, in the absence of direct comparisons, assessment of relative efficacy, safety, and tolerability therefore needs to be based on indirect comparisons ideally based on network meta-analytic methods as utilized in this study. The main limitation of a network meta-analysis in this setting is that unlike in individual patient data analysis where the unit of analysis is an individual study participant, in a meta-analysis, the unit of analysis is each individual trial. With only seven trials included, statistical power is reduced and this may decrease the certainty of our analysis.

In summary, despite differences between trial effect sizes and statistical significance, in this network meta-analysis, there was no statistically significant difference in OS between the different CDK4/6 inhibitors. Significant differences between CDK4/6i were observed for safety and tolerability outcomes. Real-world data analyses may help to identify if a there is a meaningful inter-drug difference in efficacy, safety or tolerability.

### Supplementary Information

Supplementary Information.

## Data Availability

All data available upon request to corresponding author, E. Amir.
