# Supplementary material for: Comparative overall survival of CDK4/6 inhibitors in combination with endocrine therapy in advanced breast cancer
Source: Sci Rep. 2024 Feb 7;14:3129. doi: 10.1038/s41598-024-53151-8 (PMC10850180; doi:10.1038/s41598-024-53151-8)

**APPENDIX**

**Control palbociclib with fulvestrant**


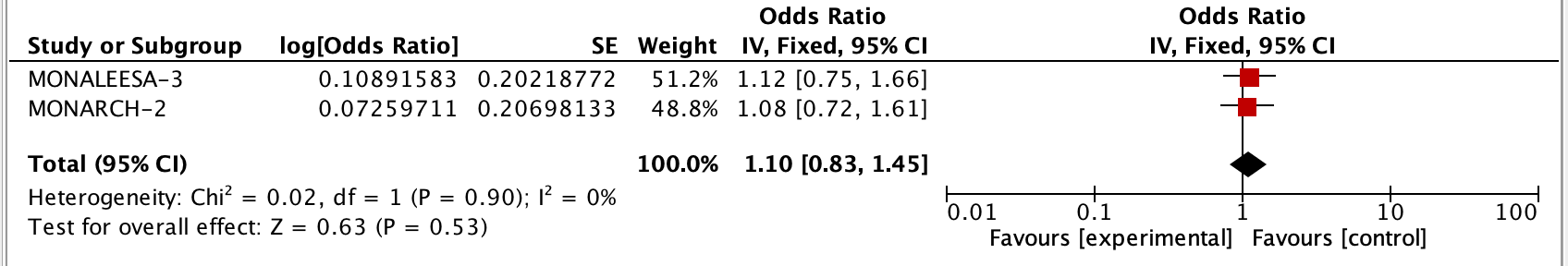


**Control palbociclib with AI/tamoxifen** **
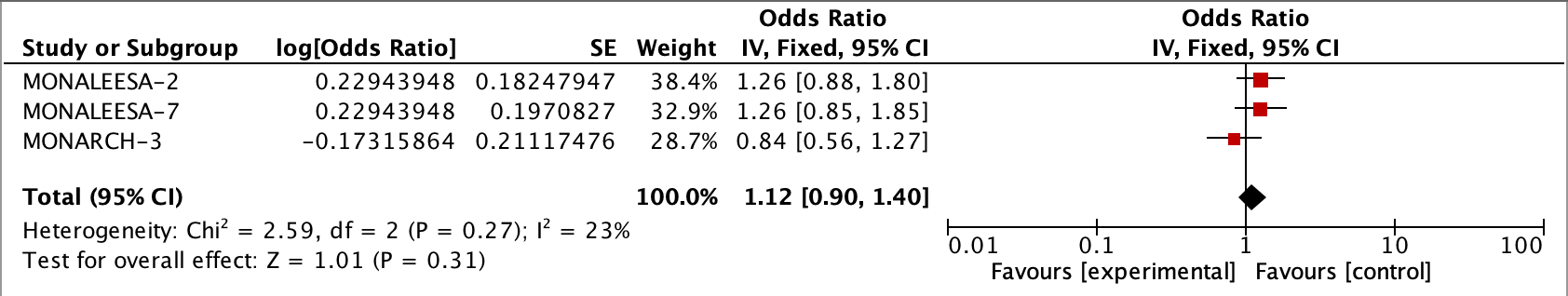
**

**Control ribociclib with fulvestrant**


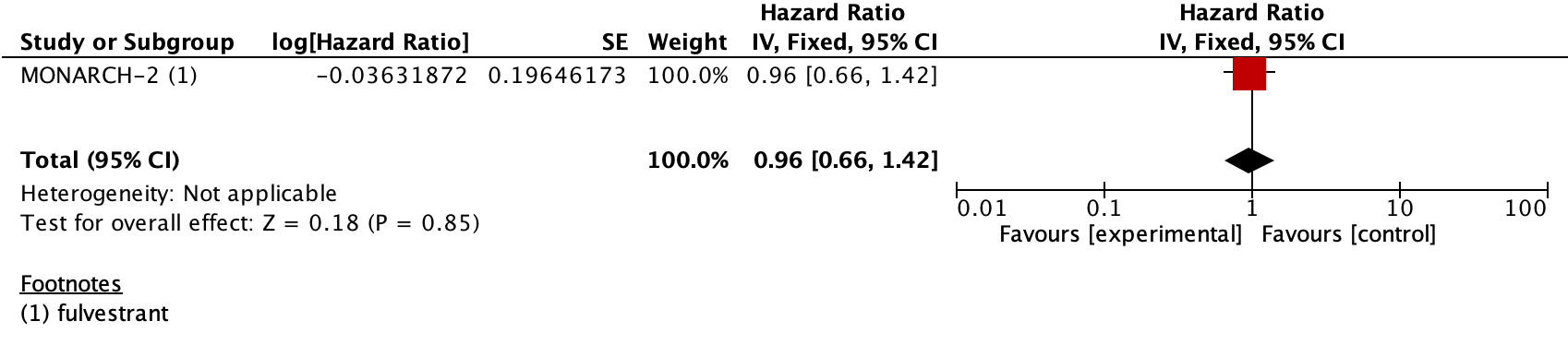


**Control abemaciclib with AI/tamoxifen**


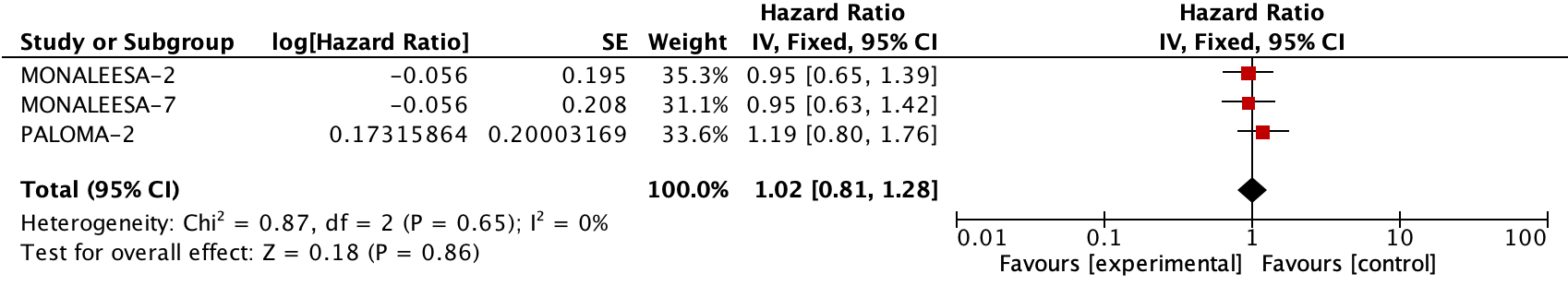


**Control abemaciclib with fulvestrant**
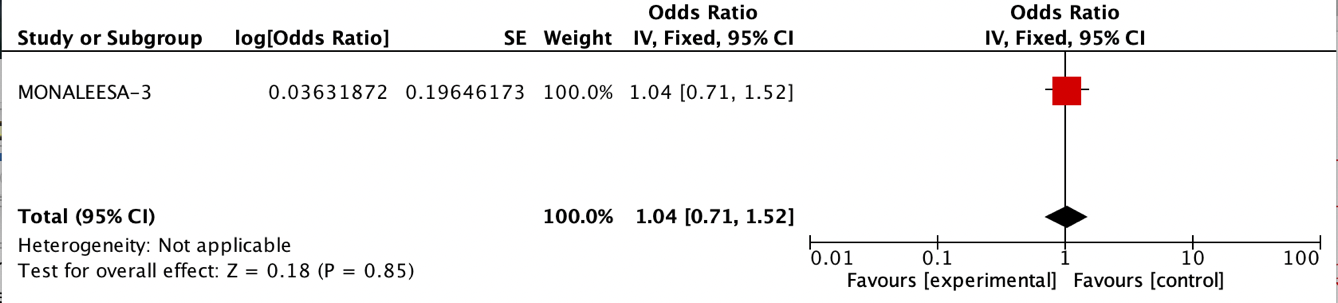


Palbociclib (control) versus Abemaciclib with fulvestrant side effect profile

(MONARCH 2 vs PALOMA-3)

**
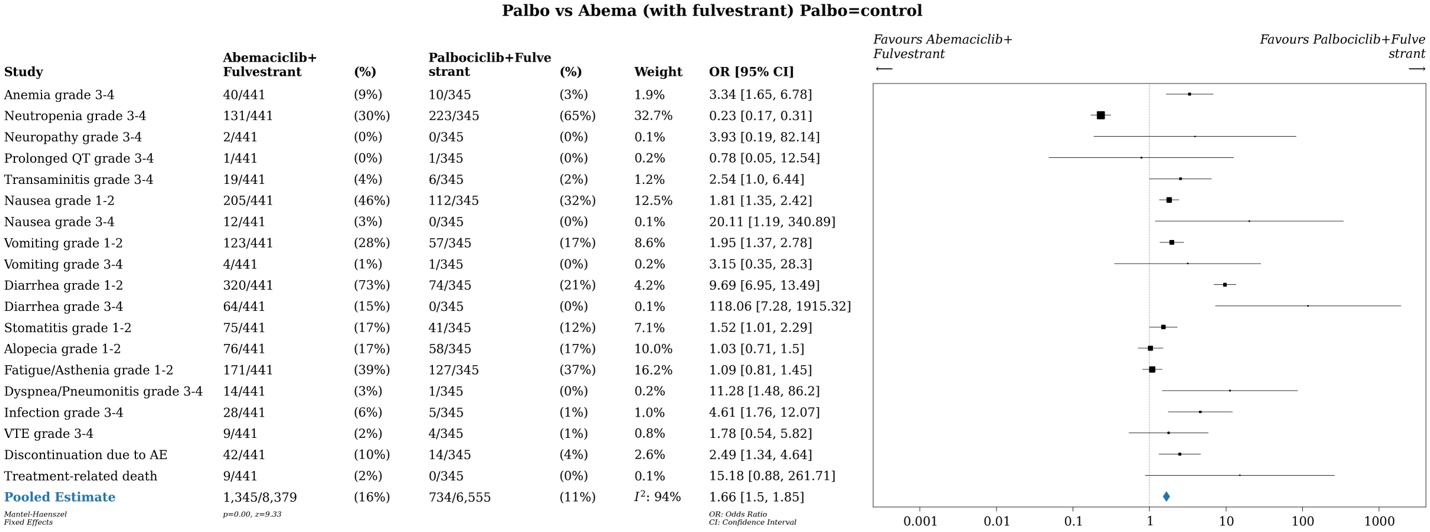
**

Palbociclib (control) vs Abemaciclib with AI side effect profile

(MONARCH 3 and PALOMA-2)


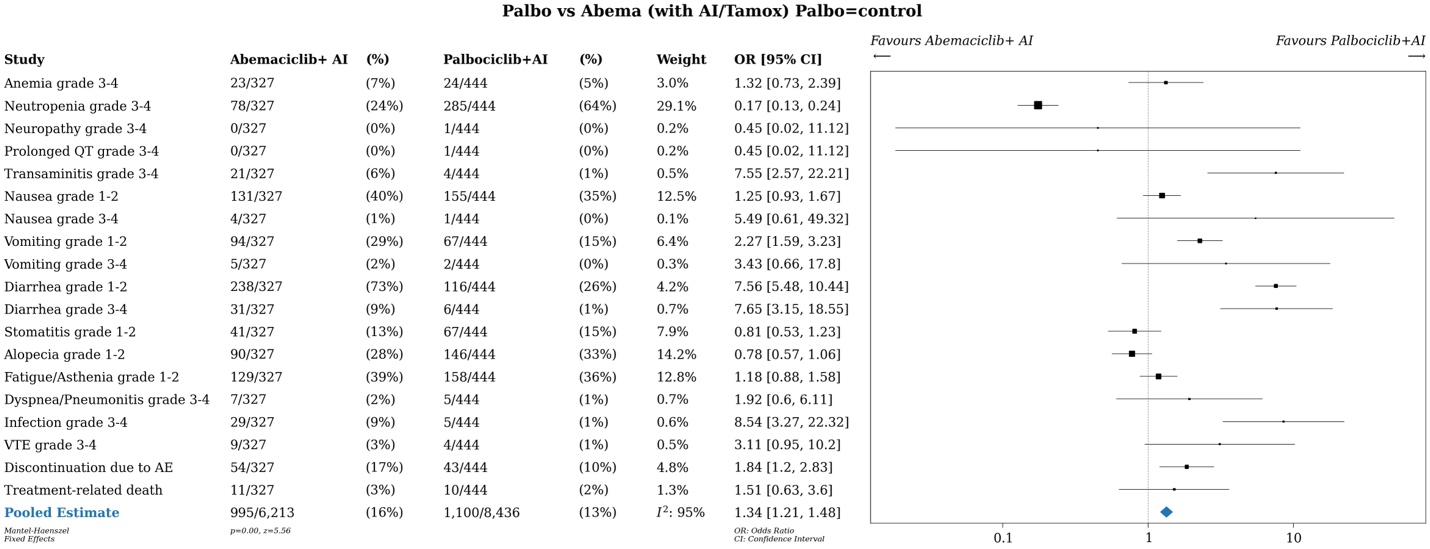


Palbociclib (control) vs Ribociclib with fulvestrant side effect profile

(MONALEESA 3, PALOMA 3)


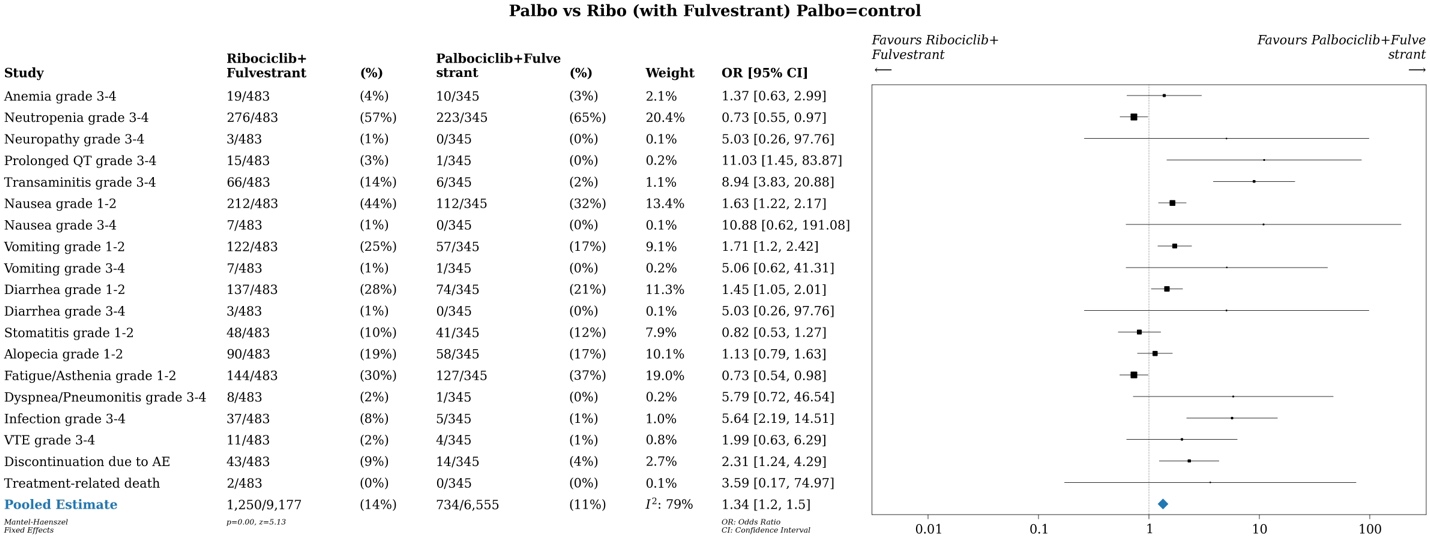


Palbociclib (control) vs Ribociclib with AI/tamoxifen side effect profile (PALOMA-3 vs MONALEESA-2 and -7 (combined))


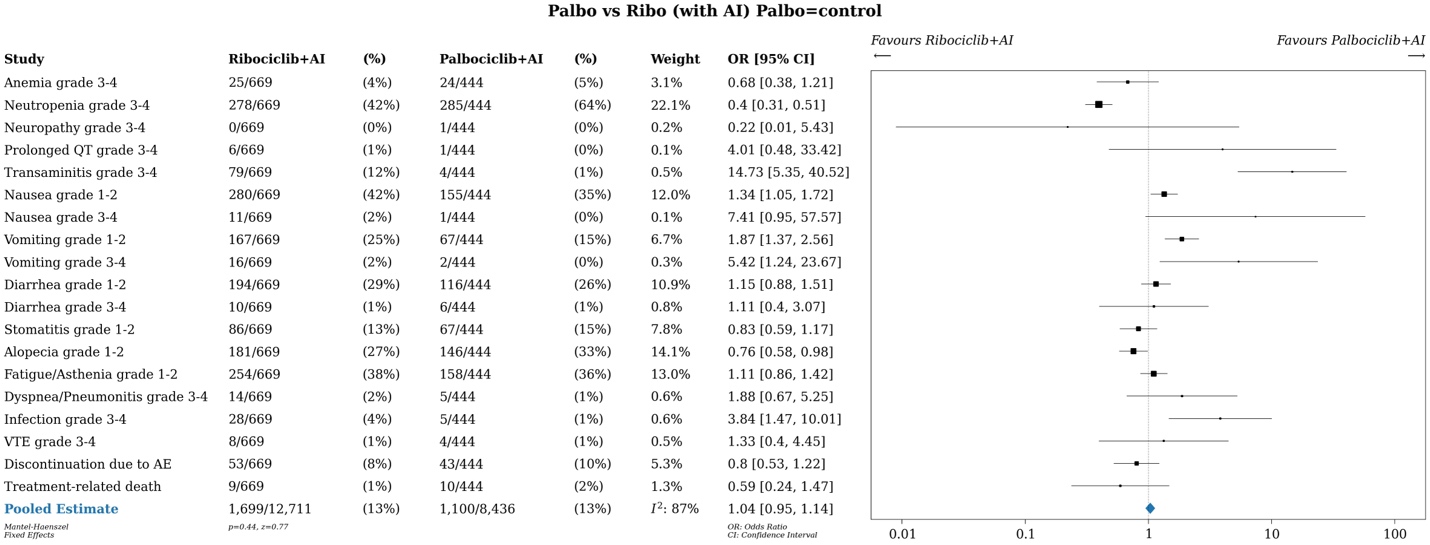


Ribociclib (control) vs Abemaciclib with fulvestrant side effect profile

(MONALEESA 3, MONARCH 2)


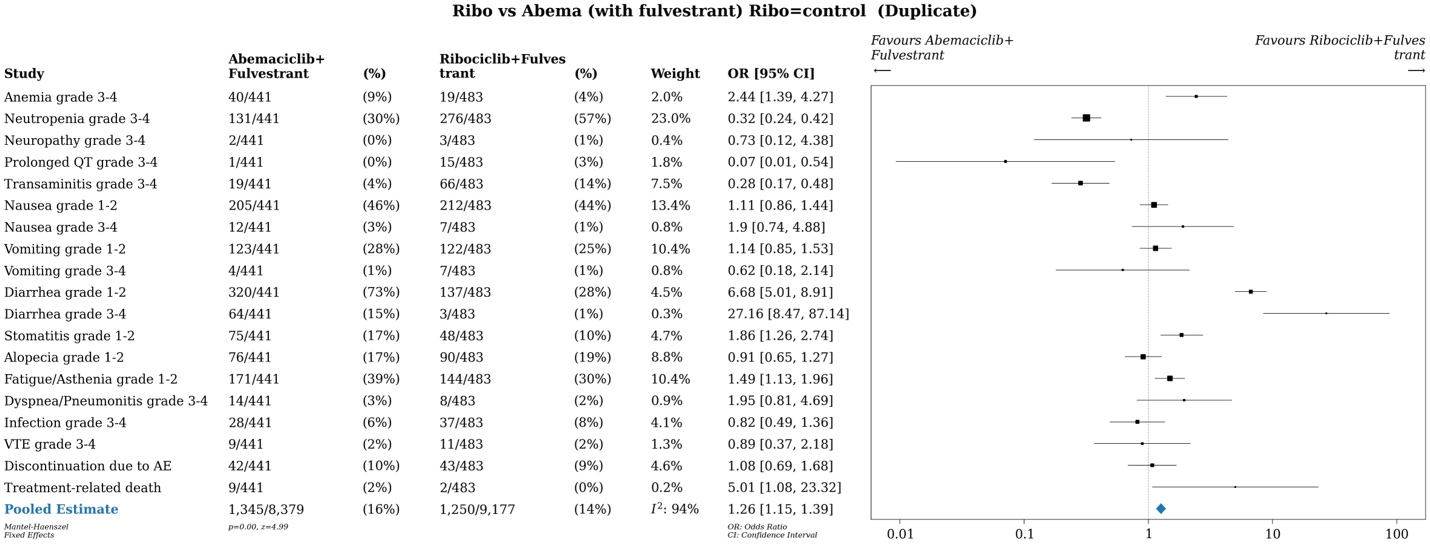


Ribociclib (control) vs Abemaciclib with AI/tamoxifen side effect profile

(MONARCH 3 vs MONALEESA-2 and -7 (combined))


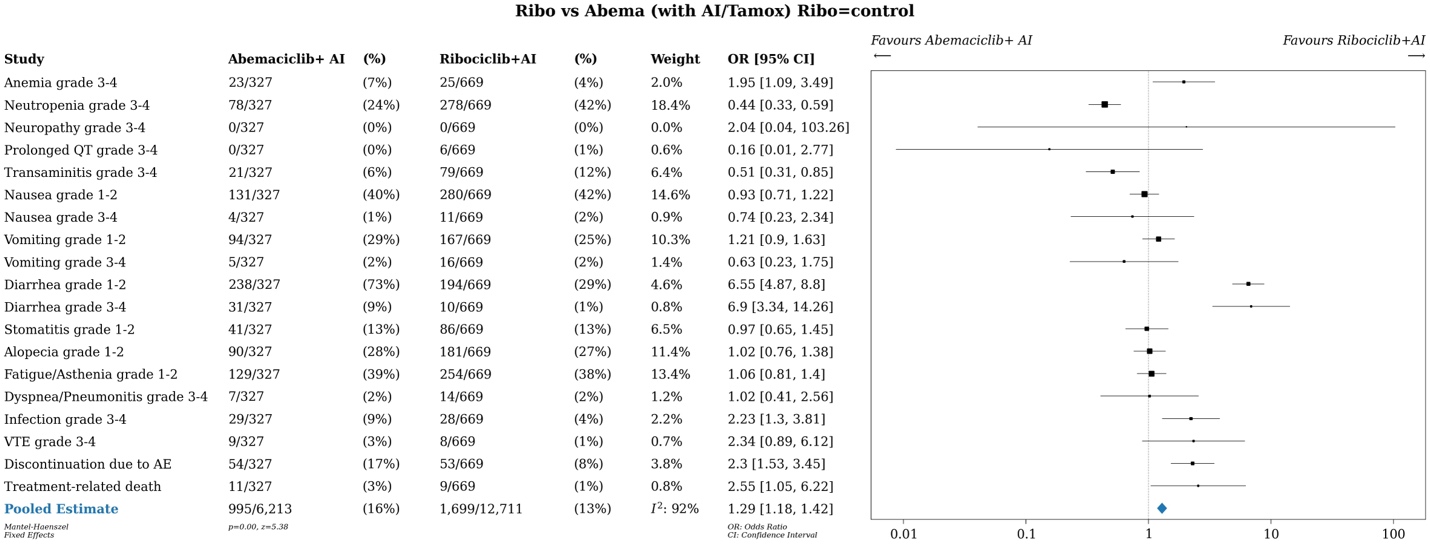


Ribociclib (control) vs Abema discontinuation


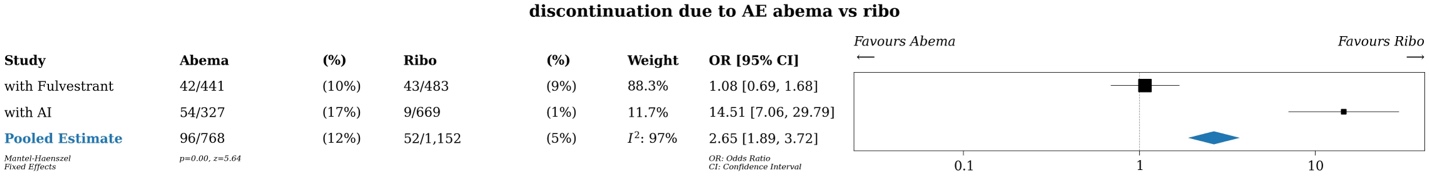


Ribociclib (control) vs Abema death due to AE


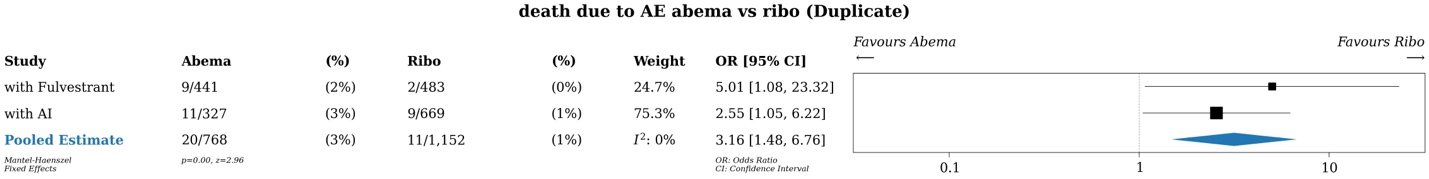


Palbociclib (control) vs Ribo discontinuation


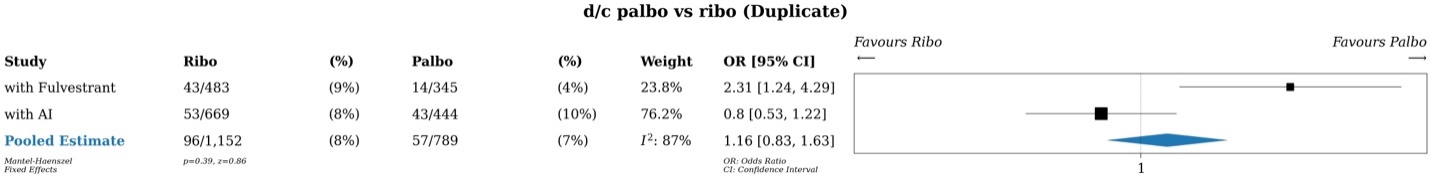


Palbociclib (control) vs Ribo death due to AE


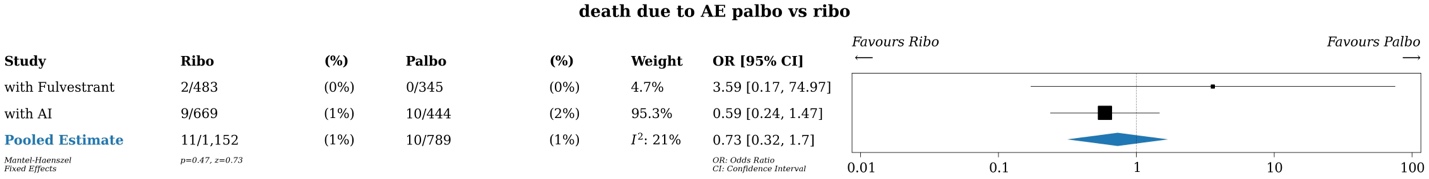


Palbociclib (control) vs Abema discontinuation


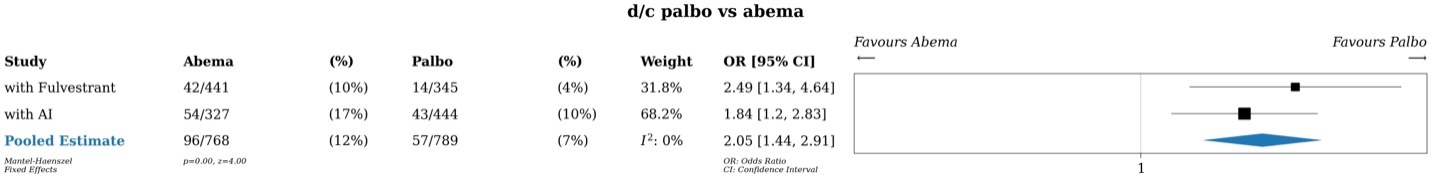


Palbociclib (control) vs Abema death due to AE


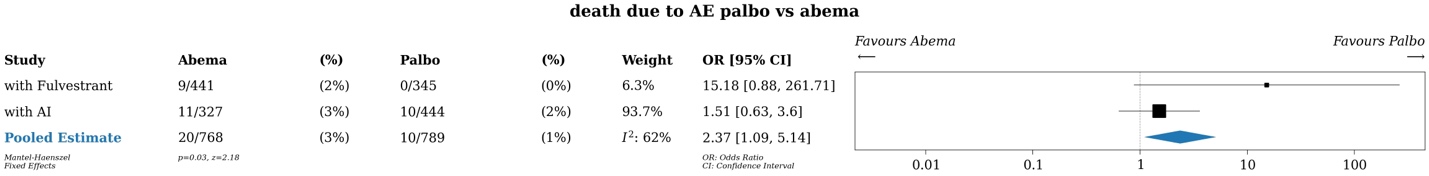

Supplement: Supplementary file 1 — Supplementary Information. [file 41598_2024_53151_MOESM1_ESM.docx]
